# The explosive trinitrotoluene (TNT) induces gene expression of carbonyl reductase in the blue mussel (*Mytilus* spp.): a new promising biomarker for sea dumped war relicts?

**DOI:** 10.1007/s00204-020-02931-y

**Published:** 2020-10-22

**Authors:** Jennifer S. Strehse, Matthias Brenner, Michael Kisiela, Edmund Maser

**Affiliations:** 1 Institute of Toxicology and Pharmacology for Natural Scientists, University Medical School Schleswig-Holstein, Brunswiker Str. 10, 24105 Kiel, Germany; 2 Alfred Wegener Institute Helmholtz Centre for Polar and Marine Research, Am Handelshafen 12, 27570 Bremerhaven, Germany

**Keywords:** Dumped munitions, Blue mussels, Short-chain dehydrogenases reduactases (SDR), Carbonyl reductase, Biomarker, Oxidative stress

## Abstract

Millions of tons of all kind of munitions, including mines, bombs and torpedoes have been dumped after World War II in the marine environment and do now pose a new threat to the seas worldwide. Beside the acute risk of unwanted detonation, there is a chronic risk of contamination, because the metal vessels corrode and the toxic and carcinogenic explosives (trinitrotoluene (TNT) and metabolites) leak into the environment. While the mechanism of toxicity and carcinogenicity of TNT and its derivatives occurs through its capability of inducing oxidative stress in the target biota, we had the idea if TNT can induce the gene expression of carbonyl reductase in blue mussels. Carbonyl reductases are members of the short-chain dehydrogenase/reductase (SDR) superfamily. They metabolize xenobiotics bearing carbonyl functions, but also endogenous signal molecules such as steroid hormones, prostaglandins, biogenic amines, as well as sugar and lipid peroxidation derived reactive carbonyls, the latter providing a defence mechanism against oxidative stress and reactive oxygen species (ROS). Here, we identified and cloned the gene coding for carbonyl reductase from the blue mussel *Mytilus* spp. by a bioinformatics approach. In both laboratory and field studies, we could show that TNT induces a strong and concentration-dependent induction of gene expression of carbonyl reductase in the blue mussel. Carbonyl reductase may thus serve as a biomarker for TNT exposure on a molecular level which is useful to detect TNT contaminations in the environment and to perform a risk assessment both for the ecosphere and the human seafood consumer.

## Introduction

Trinitrotoluene (TNT) is one of the most used explosives worldwide, nowadays and in the past, both in civil and military activities (Juhasz and Naidu 2007; Beck et al. 2018). Since World War I, military munitions have been entered into the marine environment through two different ways: First, during war actions like fired but not exploded ammunition or munitions as a load on sunken vessels and shot down fighter aircrafts (Beck et al. 2018); second, by sea-dumping of munitions after World Wars I and II, since the involved nations were faced with a nearly uncountable number of ammunitions and other explosive materials produced by themselves or leftovers from the defeated nations (Beck et al. 2018). Today, as a consequence of these actions, we have to face several problems with regard to munitions in the seas. In addition to the risk of unwanted explosions, the most serious problem is that due to the corrosion of the munitions housings, toxic chemicals are being released and enter the marine environment. Porter et al. (2011) detected TNT and other explosive chemicals not only in sediment and water but also in biota such as for example in different kinds of corals, dusky damselfish (*Stegastes adustus*) and sea urchin (*Diadema antillarum*) collected at the naval gunnery and bombing range of Isla de Vieques, Puerto Rico.

TNT is toxic to all organisms. In humans, TNT and metabolites are mutagenic and of relevant urothelial carcinogenicity as well as toxic to the liver, kidneys, eyes, skin, blood system and spleen (Bolt et al. 2006). Many studies have shown acute and chronic toxic effects on marine species like shrimp, fish, copepods, corals, amphipods and bivalves (Nipper et al. 2001; Rosen and Lotufo 2007; Ek et al. 2008; Lotufo et al. 2016). Recently, Koske et al. (2019) proofed the genotoxicity of TNT using zebrafish embryos (*Danio rerio*). Schuster et al. (2020) demonstrated negative multilevel biological effects in the Baltic blue mussel (*Mytilus* spp.). Interestingly, the mechanism of toxicity and carcinogenicity of TNT and its derivatives occurs through its capability of inducing oxidative stress and reactive oxygen species (ROS) in the target biota (Bolt et al. 2006). In HepG2 and HepG3 cells, ROS formation by TNT causes DNA damage and induces apoptosis (Liao et al. 2017). Another study demonstrated that TNT induces oxidative stress in mouse, rat and human hepatoma cells (Naumenko et al. 2017).

Oxidative stress is able to perform peroxidation reactions of lipoproteins and lipids, thereby producing reactive carbonyl compounds which lead to modifications of DNA, lipids and proteins (Esterbauer et al. 1991). Detoxification by enzymatic carbonyl reduction of reactive carbonyl compounds to their corresponding alcohols, therefore, is a well-working defence mechanism. This reaction is catalysed by enzymes from two different protein superfamilies, referred to as aldo–keto reductases (AKR) (Penning 2015) and short-chain dehydrogenases/reductases (SDR) (Persson et al. 2009).

A subgroup of the SDRs, the carbonyl reductases, are of particular importance in the defence against oxidative stress and are particularly dealt with in this publication. Human carbonyl reductase 1 (CBR1; SDR21C1) was first characterized by Wermuth (1981). Later, human carbonyl reductase 3 (CBR3; SDR21C2) was identified by Watanabe et al. (1998). While neurodegenerative disorders in humans like Parkinson’s, Alzheimer’s, Creutzfeld-Jakob and Huntington’s diseases are obviously aggravated by oxidative stress in the brain (Sayre et al. 2001; Dawson 2003), human CBR1 may contribute to neuroprotection due to its ability to metabolize reactive carbonyls derived from lipid peroxidation (Maser 2006). The role of CBR1 and CBR3 in the protection against ROS is underlined by their inducibility under conditions of oxidative stress (Ebert et al. 2010; Miura et al. 2013). In addition, toxic chemicals with impact on the environment like paraquat and even treated sewage are able to increase carbonyl reductase expression in *Oncorhynchus mykiss* and *Zoarces viviparus* (Albertsson et al. 2010, 2012).

**Fig. 1:**
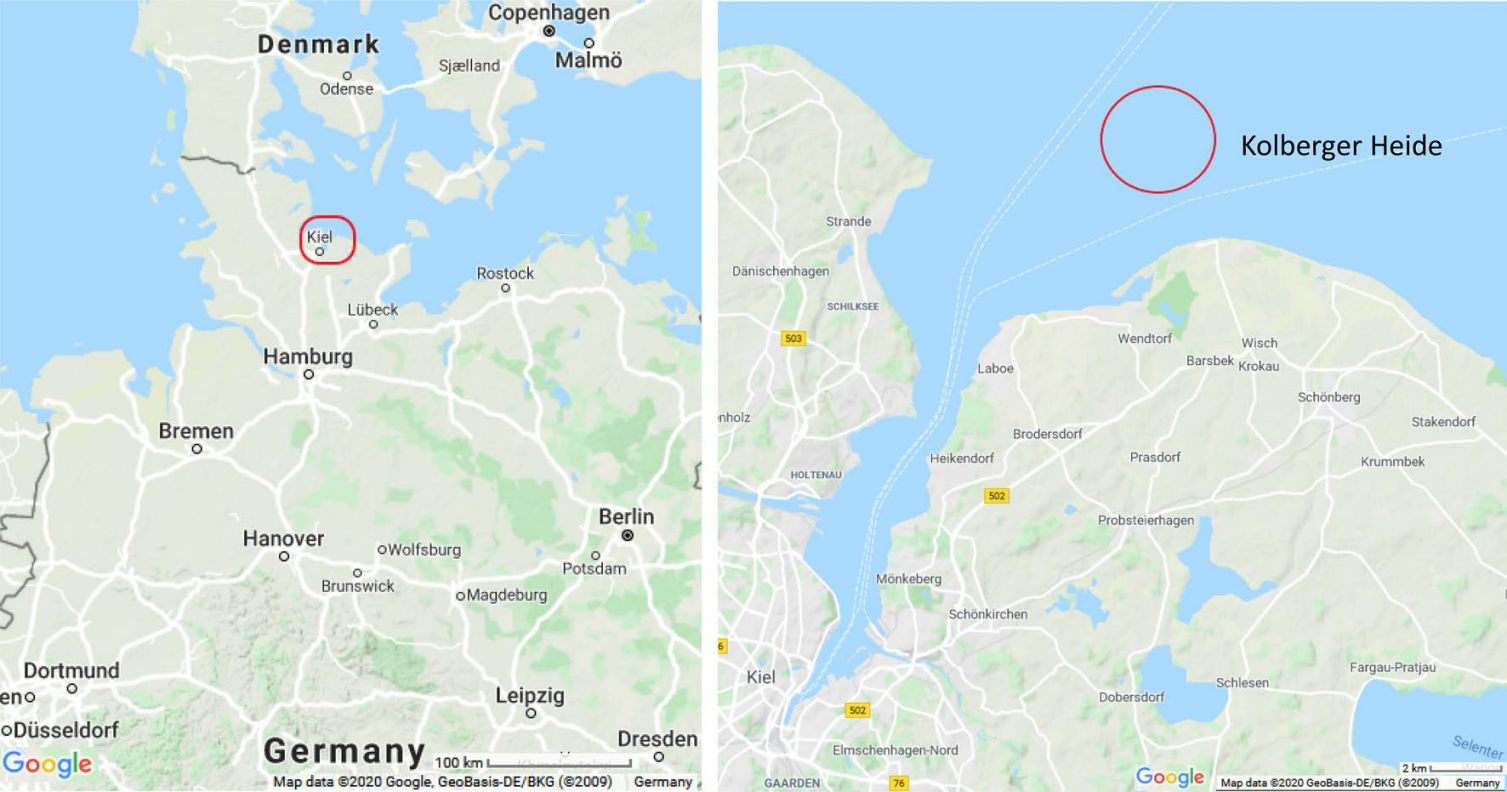
Location of the study site Kolberger Heide at the entrance to Kiel Fjord in northern Germany in the western Baltic Sea (source: www.google.de/maps)

In previous investigations, TNT and its metabolites 2-amino-4,6-dinitrotoluene (2-ADNT) and 4-amino-2,6-dinitrotoluene (4-ADNT) were detected in blue mussels (*Mytilus* spp.) which had been exposed for several weeks in three active biomonitoring studies at Kolberger Heide (Strehse et al. 2017; Appel et al. 2018; Maser and Strehse 2020) (Fig. 1). Bivalves, as filter-feeding organisms are able to concentrate chemicals in their tissues, are robust and survive under moderate levels of different pollutants (Farrington et al. 2016). In many biomonitoring studies, mussels are, therefore, used as bioindicators for environmental pollution (Strehse and Maser 2020). On the other hand, with regard to marine pollution by TNT and its metabolites, specific biomarkers are urgently sought to detect TNT contaminations in the environment and to perform a risk assessment both for the ecosphere and the human seafood consumer.

Based on these facts that TNT exerts its toxicity through the development of oxidative stress and ROS, on the one side, and that the genes of carbonyl reductase are upregulated in their expression by ROS, on the other, we examined in the present approach whether the corresponding gene in the mussel is induced by TNT and whether carbonyl reductase is able to act as a specific biomarker in blue mussels for TNT detection. To answer this question, identification and cloning of the carbonyl reductase gene in *Mytilus* spp. as well as laboratory exposure studies towards TNT and field experiments were performed.

## Material and methods

### Identification and cloning of the carbonyl reductase gene in *Mytilus* spp.

**Fig. 2:**
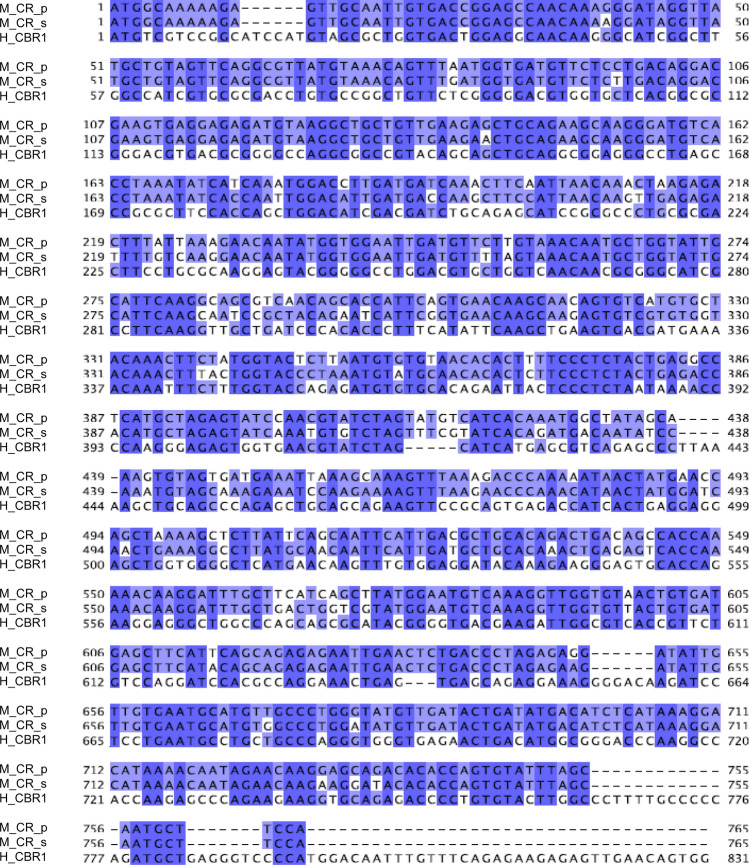
Identification and cloning of the carbonyl reductase gene in *Mytilus* spp. *M_CR_p* “proposed” sequence of the carbonyl reductase gene, *M_CR_s* sequence of the carbonyl reductase gene after cloning and verification by Sanger sequencing, *H_CBR1* sequence of human carbonyl reductase CBR1 from NCBI (GenBank accession number CR541708.1)

For identification and cloning of the *Mytilus* spp. carbonyl reductase gene and to obtain the optimum primer sequences for its amplification a BLAST search for carbonyl reductase genes on *Mytilus* genomic data and various genomes of model organisms was performed. From the resulting “proposed” gene sequence (Fig. 2) primers were designed (Table 1) to obtain the coding sequence of the carbonyl reductase gene of *Mytilus* spp. used in the present study. Of note, the *Mytilus* species occurring in the western Baltic Sea are obviously a hybrid form of *Mytilus trossulus* and *Mytilus edulis* (Stuckas et al. 2017).

**Table 1:** Primers designed for RT-PCR

Primer name	GenBank accession no	Forward primer (5′–3′)	Reverse primer (5′–3′)	Amplicon size (bp)
CR	^a^	ATGGCAAAAAGAGTTGCAATTGTG	TGGAAGCATTGCTAAATACACTGGTG	765
18 s RNA	L33448	CAACCTGGTTGATCCTGCCAGT	TATTGGAGCTGGAATTACCGCGG	605

^a^No accession number for the “proposed” sequence of carbonyl reductase (CR) is given

To verify the bioinformatical outcome Sanger sequencing was performed. In brief: for amplification of the carbonyl reductase gene the coding sequence was cloned into a pJET 1.2/blunt cloning vector. An amount of 1.5 µL cDNA was amplified with Phusion Hot Start II DNA polymerase (Thermo Fisher Scientific, Waltham, MA, USA), 20 pmol of primers as described in Table 1, and 200 µM dNTPs in a total of volume 20 µL. Thermo cycling was performed using the following conditions: initial denaturation at 98 °C for 30 s followed by 35 amplification cycles (98 °C for 10 s, 50 °C for 30 s, 72 °C for 30 s) and a final extension step at 72 °C for 10 min. The PCR product (765 bp) was cloned into a pJET 1.2/blunt cloning vector using the CloneJET PCR Cloning Kit (Thermo Fisher Scientific, Waltham, MA, USA) and NEB 5-alpha Competent *E. coli* (New England BioLabs Inc., Ipswich, MA, USA). The carbonyl reductase gene obtained from *Mytilus* spp. was verified by Sanger sequencing at the Institute of Clinical Molecular Biology, Kiel, Germany (Fig. 2).

### Laboratory exposure experiments

Blue mussels (*Mytilus* spp.) were purchased from a commercial mussel farm in Kiel Fjord. The mussels were transferred to the laboratory in an aerated cooling box with ambient seawater eight days before the experiments started. At the farm, water temperature and salinity were measured and recorded. Corresponding to the measured temperature, mussels were kept in a temperature-controlled lab using two 20-L aquaria filled with ambient seawater from the farm site. After 48 h, ambient seawater was exchanged by half of the volume using premixed artificial seawater with an equal to ambient salinity of 18 psu. This procedure was repeated every 24 h until the experiments started.

The artificial seawater was produced daily by mixing deionized water and Coral Pro Salt (Red Sea, Düsseldorf, Germany), and pre-cooled before usage. The salinity was checked before water exchange using a conductivity meter. Every 48 h, mussels were fed with 6 ml live phytoplankton (Premium Reef Blend) per aquarium two hours prior to the water exchange. Twenty-four hours before the start of the exposure experiments, mussels were transferred to 15-L aquaria filled with 10 L of artificial seawater. During exposure, the room was kept constantly at 9 °C (96 h exposure) and 3 °C (21 days exposure), with a light/dark rhythm of 8:16 according to the ambient conditions at the mussel farm.

Three aquaria were used for each of the treatment concentrations and seven mussels were exposed per aquarium. The artificial seawater in the aquaria was spiked with different concentrations of a TNT solution, except for the three control aquaria. Water exchange and re-dosing of dissolved TNT was conducted on a daily basis by emptying and refilling the aquaria. To yield the required TNT concentration in the artificial seawater, a corresponding amount of TNT solution was transferred from the stock solution to the aquaria. The stock solution of 100 mg/L TNT was produced by the GEKA mbH (Munster, Germany) by dissolving TNT crumbs received from grenades from WW II in dichloromethane. Aliquots were removed from the solution, evaporated at room temperature and re-dissolved in water at 80 °C.

The following exposure experiments were performed: (1) an acute toxicity test (96 h) with aquaria concentrations of 0; 0.31; 0.62; 1.25; 2.5, 5; 10 mg/L TNT solution following the guidelines of the OECD (2019) and (2) a 21-day lasting chronic exposure experiment using 0; 0.31; 0.62; 1.25; 2.5 mg/L TNT solution (OECD 2009). After the experiments, the mussels were immediately kept dry and cool until dissection.

### Field exposure experiment

The field exposure study was conducted at the munitions dumpsite Kolberger Heide in Kiel Bight at the entrance to Kiel Fjord, Germany (Fig. 1). In this area, several torpedo heads and mines had been destroyed selectively by blast-in-place operations in recent years (Böttcher et al. 2011). As a result, explosive materials have been distributed in the adjacent environment on the seafloor (Strehse et al. 2017). Two mussel bags with 15 mussels each (60.5 mm ± 6.0 mm length), were placed in January 2018 close to blast craters with explosive materials in the vicinity in a depth of approximately 11 m. One of these bags was placed at the ground directly adjacent to a chunk of explosive material (7 U), the other at a height of 1 m (7 O). The exposure period amounted to 58 days and the sampling was done by Kiel University research divers. After recovery the mussels were immediately placed on dry ice and stored at − 80 °C until further use.

### Tissue isolation, RNA extraction and cDNA synthesis

Mussels were opened and a maximum of 20 mg tissue of each, gills, hepatopancreas, mantle and muscle, were transferred into 1.5 mL Eppendorf tubes. Up to three samples of each tissue and mussel were taken. For stabilization of RNA, 200 µL of RNA*later*^®^ reagent (Qiagen, Venlo, Netherlands) were added to each tube and incubated overnight. Tissues were then removed from the reagent, transferred to 2 mL Eppendorf tubes and stored until further processing at − 80 °C. RNA was isolated using the commercially available Roti^®^-Prep RNA MINI kit (Carl Roth, Karlsruhe, Germany). After addition of lysis buffer, the tissues were homogenized using a T25 Ultra-Turrax (Ika Works Inc., Staufen im Breisgau, Germany). RNA was purified according to the manufacturer’s protocol and quantified with Qubit^®^ HS RNA Assay kit (Carlsbad, CA, USA) on a Qubit^®^ 2.0 Fluorometer (Carlsbad, CA, USA). RNA was stored at − 80 °C until further use. An amount of 1000 ng of RNA was reverse transcribed using RevertAid H minus reverse transcriptase (Thermo Fisher, Waltham, MA, USA) with 400 ng of Random Primers (Promega, Madison, WI, USA) for 10 min at 25 °C, followed by 60 min at 42 °C and at − 20 °C.

### Gene expression experiments by reverse transcription-polymerase chain reaction (RT-PCR)

PCR was performed with Phire Green Hot Start II DNA polymerase (Thermo Fisher Scientific, Waltham, MA, USA) using 1.5 µL cDNA, 20 pmol primers and 200 µM dNTPs in a total volume of 20 µL. Thermo cycling was performed using the following conditions: initial denaturation at 98 °C for 30 s followed by varying amplification cycles (six for the housekeeping gene and 22–29 cycles for carbonyl reductase, respectively). The temperature program during the amplification cycles was performed at 98 °C for 5 s, 50 °C for 5 s, 72 °C for 15 s. A final extension step was performed at 72 °C for 1 min*. M. edulis* 18 s RNA served as housekeeping gene (Cubero-Leon et al. 2012). An amount of 18 µL of amplified PCR products was separated electrophoretically on 1% agarose gels and visualized by staining with GelRed™ (Biotest, Mannheim, Germany). Gels were documented digitally with Intas® Gel-imaging system (Intas, Göttingen, Germany) and densitometrically analysed with GIMP 2.8 software.

### Determination of explosive chemicals in mussel tissues

The chemical tissue analysis was performed as described in Strehse et al. (2017): Mussel tissues were thawed, whole meat was placed in a 50 mL polypropylene tube per mussel, and homogenized using a T25 Ultra-Turrax (Ika Works Inc., Staufen im Breisgau, Germany). Tissues were aliquoted into 1.0 g portions in 50 mL polypropylene tubes, and 5 mL gradient grade acetonitrile (Th. Geyer GmbH & Co. kg, Renningen, Germany) were added per tube. Each sample was mixed for one minute using a VF2 vortex mixer (Ika Works Inc., Staufen im Breisgau, Germany). Tubes were centrifuged for 5 min at 4 100 rpm (20 °C) with a Heraeus Megafuge 11R centrifuge (Thermo Fisher Scientific Inc., Waltham, MA, USA). Supernatants were decanted and filled with acetonitrile to a total volume of 10.0 mL. GC/MS-MS analysis was performed on a TSQ 8000 EVO Triple Quadrupole Mass Spectrometer (Thermo Fisher Scientific Inc., Waltham, MA, USA) with a Trace 1310 Gas Chromatograph (Thermo Fisher Scientific Inc., Waltham, MA, USA) and using a TraceGOLD™ TG-5SilMS GC column (30 m × 0.25 mm × 0.25 µm).

### Data analysis and statistics

Values of the measured concentrations of TNT and the metabolites 2-ADNT and 4-ADNT in the mussel tissues are expressed as mean ± standard deviation. Descriptive statistics of the densitometric results obtained from the RT-PCR experiments were computed with GraphPad Prism 8 (version 8.4.2).

## Results

### Identification and cloning of the carbonyl reductase gene in *Mytilus* spp.

Since the *Mytilus* species occurring in the Baltic Sea are obviously a hybrid form of *Mytilus trossulus* and *Mytilus edulis*, we aimed, at first, on the identification and cloning of the carbonyl reductase gene of the *Mytilus* species used in the present study. By a bioinformatics approach, we obtained a “proposed” carbonyl reductase gene sequence (Fig. 2) which was used for primer design and cloning of the “true” coding sequence of the carbonyl reductase gene for our study (Fig. 2). The confirmed *Mytilus* spp. carbonyl reductase gene shares 89% identity to the “proposed” primary structure and 56% to human CBR1 (Fig. 2). Based on the latter fact, the *Mytilus* spp. carbonyl reductase can be regarded as a functional homolog to human CBR1, because, in addition, it shares all the essential SDR motifs (Persson et al. 2009).

### Basal expression of carbonyl reductase in *Mytilus* spp. tissues

**Fig. 3:**
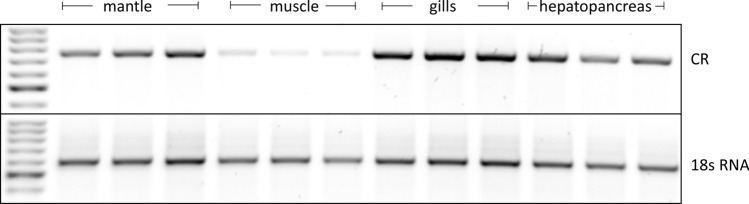
Basal expression of the carbonyl reductase in *Mytilus* spp. tissues. Tissue samples from mantle, muscle, gills and hepatopancreas of control mussels (without TNT exposure) were processed for mRNA extraction. Primers used for RT-PCR are shown in Table 1. 18 s RNA served as the housekeeping gene

As a reference for the extent of gene induction through TNT, the basal expression of the carbonyl reductase gene was examined in different tissues of *Mytilus* spp. without TNT exposure (controls). Mussels were dissected to obtain the following tissues of interest: mantle, muscle, gills and hepatopancreas. While muscle tissue contained the lowest levels of carbonyl reductase expression, and intermediate levels were observed for mantle tissue and the hepatopancreas, the highest expression was observed in the gills (Fig. 3). This is an interesting finding and supports the fact that gills, as the first point of contact with oxygen-rich water, are particularly exposed to oxidative stress such that there is the need for high carbonyl reductase expression.

### Carbonyl reductase gene induction in *Mytilus* spp. after 96-h TNT lab exposure

**Fig. 4:**
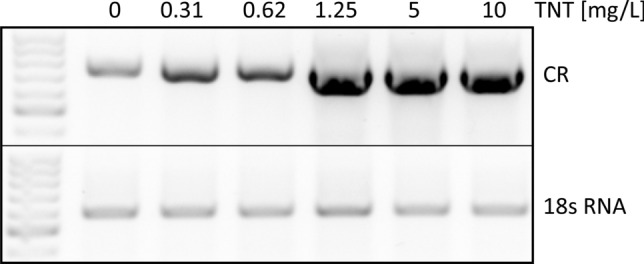
Induction of the carbonyl reductase gene in *Mytilus* spp. gills after a 96-h treatment with TNT—representative agarose gel. Mussels were exposed to increasing concentrations of TNT (ranging from 0.31 to 10 mg/L) for 96 h in laboratory aquarium studies. Exposed mussels plus control specimens (without TNT exposure) were dissected and tissues samples were processed for mRNA extraction. The primers used for RT-PCR are shown in Table 1. 18 s RNA served as the housekeeping gene

**Fig. 5:**
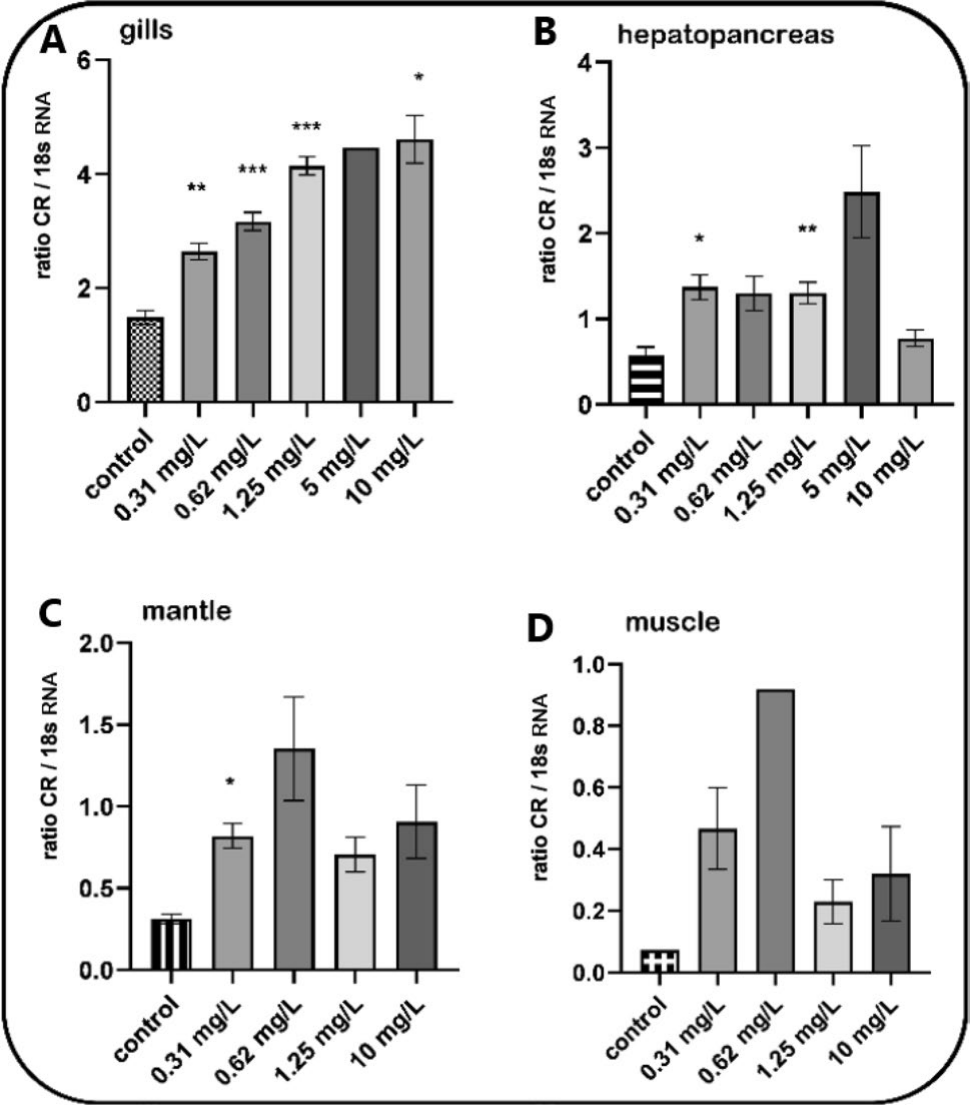
Induction of the carbonyl reductase in *Mytilus* spp. tissues after a 96-h treatment with TNT—densitometric analyses. Mussels were exposed to increasing concentrations of TNT (ranging from 0.31 to 10 mg/L) for 96 h in laboratory aquarium studies. Exposed mussels plus control specimens (without TNT exposure) were dissected and tissues samples were processed for mRNA extraction. The primers used for RT-PCR are shown in Table 1. 18 s RNA served as the housekeeping gene. Fold inductions (means with SD from *n* = 3, except muscle control with *n* = 1) were calculated from the carbonyl reductase expression relative to 18 s RNA. Statistically significant changes are indicated by asterisks (*p < 0.05, **p < 0.01, ***p < 0.001; Dunnett’s T3 multiple comparisons test. No statistical calculation for muscle was performed because of *n* = 1 in the control group)

Mussels were exposed to increasing concentrations of TNT for 96 h in laboratory aquarium studies. As shown in Figs. 4 and 5, TNT caused a concentration-dependent and statistically significant induction of the carbonyl reductase gene in the gills of *Mytilus* spp., thereby again reflecting the importance of carbonyl reductase in the gills as first-line defence against oxidative stress. Figure 4 shows a representative RT-PCR experiment (agarose gel) derived from gills with TNT concentrations ranging from 0.31 up to 10 mg/L including respective controls (without TNT exposure). Figure 5 shows the respective densitometric analyses of the RT-PCR agarose gels derived from gills, hepatopancreas, mantle and muscle resulting from the same 96 h exposure experiments. Like that in gills, the stress response towards TNT was observed in hepatopancreas, mantle and muscle tissues, but with some variability at higher concentrations compared to gills.

### Carbonyl reductase gene induction in *Mytilus* spp. after 21-day TNT lab exposure

**Fig. 6:**
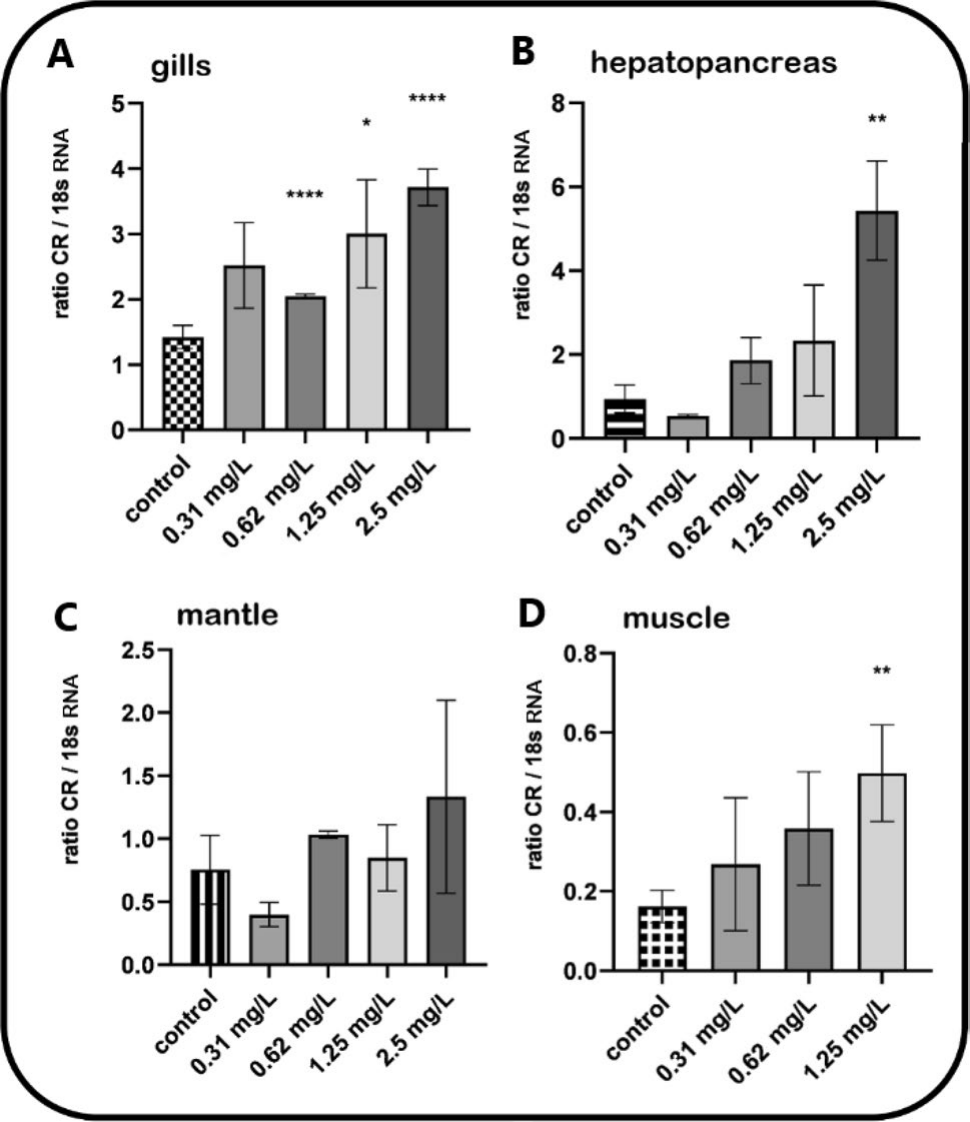
Induction of the carbonyl reductase in *Mytilus* spp. tissues after a 21-day treatment with TNT—densitometric analyses. Mussels were exposed to increasing concentrations of TNT (ranging from 0.31 to 2.5 mg/L) for 21 days in laboratory aquarium studies. Exposed mussels plus control specimens (without TNT exposure) were dissected and tissues samples were processed for mRNA extraction. The primers used for RT-PCR are shown in Table 1. 18 s RNA served as the housekeeping gene. Fold inductions (means with SD from *n* = 3–9) were calculated from the carbonyl reductase expression relative to 18 s RNA. Statistically significant changes are indicated by asterisks (*p < 0.05, **p < 0.01, ***p < 0.001, ****p < 0.0001; Dunnett’s T3 multiple comparisons test was used for the calculation of the gills and hepatopancreas results, Dunn’s multiple comparisons test for the calculation of the mantle and muscle results)

Mussels were exposed to increasing concentrations of TNT for 21 days in laboratory aquarium studies. As shown in Fig. 6, (densitometric analyses of agarose gels from RT-PCR experiments), TNT, compared to control, caused a clear induction of the carbonyl reductase gene in the gills, hepatopancreas, mantle and muscle tissues of the mussels which was highly significant in gills at 0.62 and 2.5 mg/L TNT concentrations. The lack of statistical significance in some of the columns, despite clear indications for carbonyl reductase gene induction, is due to the low amount of tissue obtained (which was a challenge upon preparation). Anyway, when looking at the y-axes it turns out that indeed gills and hepatopancreas are the tissues responsible for providing oxidative stress response.

### Field studies at Kolberger Heide–caging experiments

**Fig. 7:**
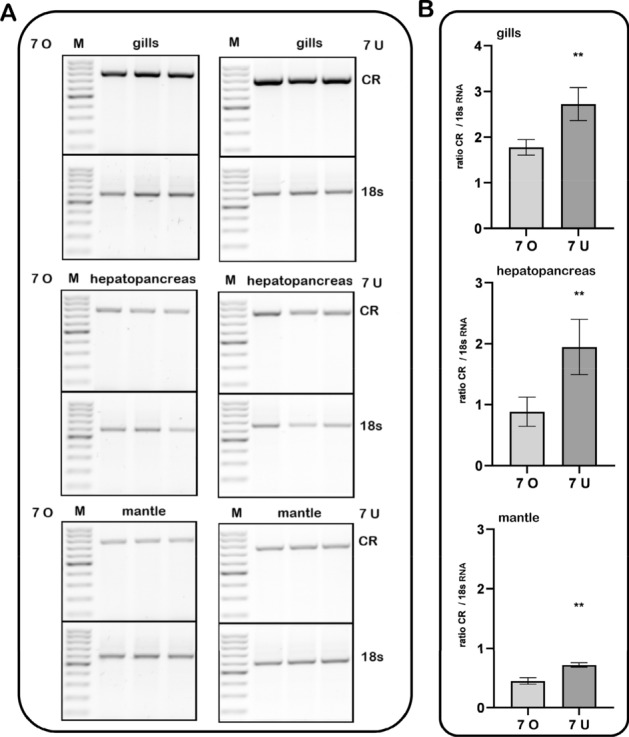
Induction of the carbonyl reductase in *Mytilus* spp. exposed in a munition dumping ground. Mussels were exposed in a field study at the ground directly adjacent to a chunk of explosive material (7 U) or at a height of one meter (7 O). After recovery (58 days exposure) mussel tissued were analysed for carbonyl reductase expression. Primers used for RT-PCR are shown in Table 1. **a** Agarose gels showing three replicates of PCR fragments per tissue; **b** Densitometric analysis of the gels shown in (**a**) (means with SD from *n* = 3). Fold inductions were calculated from the carbonyl reductase expression relative to 18 s RNA as the housekeeping gene. Statistically significant changes are indicated by asterisks (**p < 0.01; unpaired t-test with Welch’s correction)

With the help of scientific divers, mussels were exposed in mussel bags at the munitions dumping ground Kolberger Heide near a chunk of hexanite. One bag was placed at the ground directly adjacent to a chunk of explosive material (7 U), the other at a height of one meter (7 O). This is important because mussel exposure towards TNT and its metabolites could be expected to be higher at 7 U compared to 7 O (Strehse et al. 2017; Maser and Strehse 2020). Mussels were recovered after 58 days and analysed for carbonyl reductase expression. Indeed, induced gene expression of carbonyl reductase was statistically significant higher (*p* < 0.01) in 7U mussels compared to 7 O mussels (Fig. 7). Corresponding to the results from lab aquarium exposure, gills and hepatopancreas were the most responsive tissues compared to the mantle. Of note: Muscle tissue could not be obtained from any mussel.

### Occurrence of explosive chemicals in mussel tissues

All mussels of the current study were examined for their tissue content of explosive chemicals by GS/MS analysis. In all mussels from the lab exposure studies, TNT itself as well as its metabolites 2-ADNT and 4-ADNT were found, while mussels from the control group were free of detectable amounts of explosive chemicals (Table 2). Generally, in all groups values for 4-ADNT were higher than values for 2-ADNT, whereas TNT was found in only low amounts. This points to an extensive transformation of TNT to 2- and 4-ADNT within the mussels during the exposure experiments. Interestingly, the sum of TNT and metabolites determined within the mussels tissue reflects the initial TNT concentration in the aquaria, i.e. increasing TNT exposure led to higher tissue concentrations of the explosive chemicals TNT, 2-ADNT and 4-ADNT (Table 2).

**Table 2:** Mean values (± SD) of TNT, 2-amino-4,6-dinitrotoluene (2-ADNT) and 4-amino-2,6-dinitrotoluene (4-ADNT) measured in the tissue of exposed *Mytilus *spp. obtained from the 96-h and 21-day experiments

Exposure groups [mg/L]	TNT		4-ADNT		2-ADNT		∑ [ng/g]
	Mean [ng/g]	± SD	Mean [ng/g]	± SD	Mean [ng/g]	± SD	
**96-hour** exposure study							
0	n.d.		n.d.		n.d.		
0.31	25.5	12.6	659.3	160.8	508.8	119.1	1193.6
0.62	21.6	13.1	1287.1	402.7	866.4	348.2	2175.1
1.25	132.3	23.3	2473.8	606.3	2269.7	443.1	4875.8
2.5	363.2	396.4	5601.2	1813.4	5154.5	198.2	11118.9
5	594.0	578.1	7199.4	2846.9	7017.0	1244.1	14810.4
10	1469.9	115.0	30872.8	977.3	25880.9	967.6	58223.6
**21-day** exposure study							
0	n.d.		n.d.		n.d.		
0.31	30.2	4.9	622.7	129.7	313.9	49.3	966.8
0.62	41.4	20.2	1659.6	64.5	1170.4	125.5	2871.4
1.25	49.3	19.1	1290.6	774.7	917.9	658.9	2257.8
2.5	126.5	33.2	5247.1	266.5	4288.0	1874.9	9661.6

Tissue concentrations of mussels from the field studies at Kolberger Heide have already been published in Strehse et al. (2017). In brief, in mussels directly exposed at the ground near a free lying chunk of hexanite, 2-ADNT, 4-ADNT and TNT could be detected with average body burdens of 103.75 ± 12.77, 131.31 ± 9.53 and 31.04 ± 3.26 ng/g mussel wet weight, respectively. In mussels placed one meter above the ground at the same position, no TNT and 2-ADNT could be detected, but 4-ADNT was found with an average concentration of 8.71 ± 2.88 ng/g mussel wet weight. This again points to a transformation of TNT to its 2- and 4-ADNT metabolites.

### Correlation between explosive chemical exposure and carbonyl reductase gene induction

**Fig. 8:**
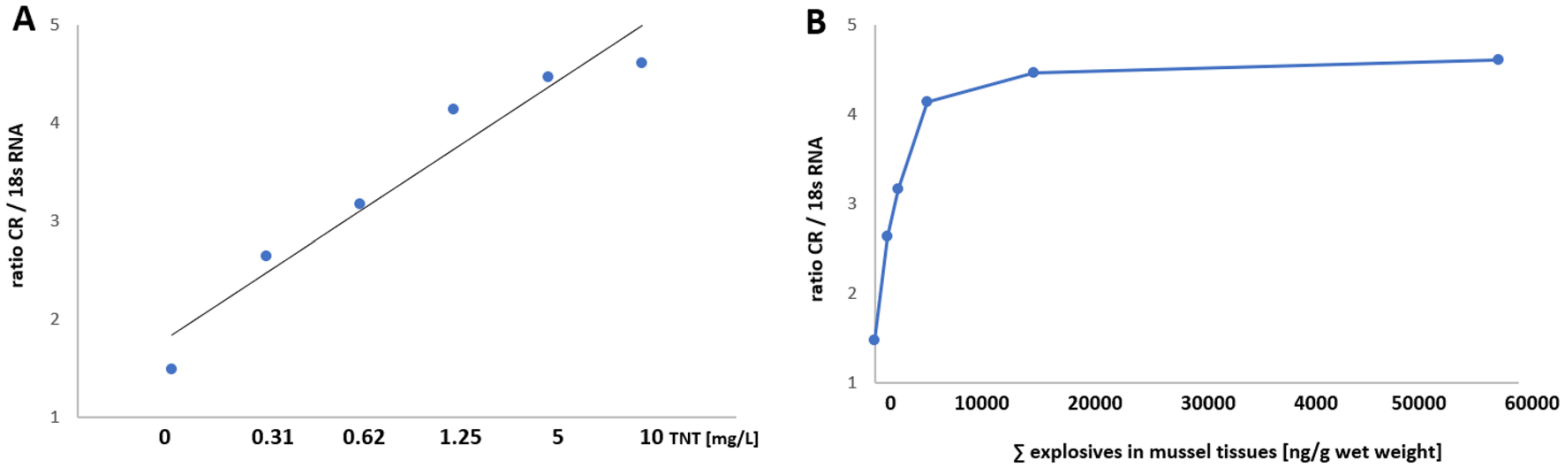
Correlation between explosive chemical exposure and carbonyl reductase gene induction. **a** Ratio of carbonyl reductase (CR) expression vs. 18 s RNA expression is plotted against initial TNT concentrations in the exposure aquaria (given in mg/L). **b** Ratio of carbonyl reductase (CR) expression vs. 18 s RNA expression is plotted against tissue concentrations of explosive chemicals (sum of TNT plus 2- and 4-ADNT) per g mussel tissue (wet weight)

Initial TNT concentrations in the exposure aquaria, given as mg/L, in most cases resulted in 3–5 fold accumulation of TNT plus metabolites in the corresponding mussels (Table 2). This finding led us to perform correlation analyses between tissue and/or water concentrations of explosive chemicals and the extent of carbonyl reductase gene induction. As shown in Fig. 8, 96 h lab exposure to TNT yielded a concentration-dependent correlation towards the ratio of carbonyl reductase gene induction versus 18 sRNA gene induction with a correlation coefficient of *R*^2^ = 0.935 in the gills. Interestingly, correlation studies considering the levels of tissue concentrations of explosive chemicals clearly showed that the maximum of carbonyl reductase gene induction occurs at around 10 µg explosives (sum of TNT plus 2- and 4-ADNT) per g mussel tissue.

## Discussion

Kolberger Heide, a section of the western Kiel Bight at the entrance to Kiel Fjord, Germany, is a warfare contaminated area with extensive research activities. This area has a size of approximately 1.260 ha and is located in a distance of three to five nautical miles to the shoreline. It was used as an area for dumping munitions after World War II (Böttcher et al. 2011). At Kolberger Heide some 30000 tons of German and British ordnance are present (Kampmeier et al. 2020). In water samples and several species like starfish (*Asteroidea *sp.), algae and tunicates collected at Kolberger Heide as well as in the bile of dab (*Limanda limanda *L.) caught in this area, TNT and other explosive residues were found (Gledhill et al. 2019; Beck et al. 2019; Koske et al. 2020).

In previous investigations, we have established the mussel *Mytilus* spp. as a biomonitoring system to determine explosive chemical contaminations leaking from corroding mines (Strehse et al. 2017; Appel et al. 2018; Maser and Strehse 2020). With this system, we could unequivocally show that explosive residues enter the marine biota in the vicinity of dumped munitions. With respect to TNT toxicity, even moderate concentrations of TNT like those found in the water column at Kolberger Heide may have an impact on the exposed mussels such as decreases in size and weight (Strehse et al. 2017; Gledhill et al. 2019). Recently, Schuster et al. (2020) demonstrated multi-level biological effects in Baltic blue mussels (*Mytilus* spp.) treated with TNT in two laboratory studies. This multi-biomarker approach comprised genetic, cellular and metabolic effects in correlation to TNT exposure were observed. Likewise, elevated levels of unspecific oxidative damages were seen. Nonetheless, neither one of the observed biomarkers alone nor the entire biomarker approach as such were able to serve as a specific indicator for a possible TNT contamination in the marine environment.

In the present investigation we, therefore, focused on the metabolic antioxidant defence system on a molecular level and concentrated on the induction of the carbonyl reductase gene which is known to respond to oxidative stress and reactive oxygen species (ROS) (Ebert et al. 2018). The existence of ROS is an integral part of human and animal life. On the one hand, ROS are actively produced within the metabolism of an organism to fulfil essential vital processes, for example in the citric acid cycle. On the other hand, xenobiotics that constantly enter living individuals can either directly be metabolized to ROS or they cause damages to cell components like DNA, proteins and other essential structures, which may contribute to the formation of ROS as well (Lushchak 2011). Fortunately, cellular organisms are able to protect themselves with the help of several non-enzymatic and enzymatic mechanisms (Ebert et al. 2018).

Here, carbonyl reducing enzymes play an important role in the defence against cellular damages caused by oxidative stress and ROS, thereby providing neuroprotection against reactive carbonyl compounds (Botella et al. 2004; Maser 2006; Ellis 2007). For example, human carbonyl reductase 1 (CBR1; SDR21C1) was first identified in the human brain (Wermuth 1981) and later shown to play a role in the protection against lipid peroxidation derived reactive aldehydes (Doorn et al. 2004; Maser 2006), thereby constituting a defence mechanism against oxidative stress in general and carbonyl stress in more detail (Ebert et al. 2018). Later, human carbonyl reductase 3 (CBR3; SDR21C2) was characterized (Watanabe et al. 1998). Interestingly, the model organism *Drosophila melanogaster* has no carbonyl reductase gene but an unrelated gene called *sniffer* (Botella et al. 2004) which was shown to provide protection against carbonyl stress in *Drosophila* (Martin et al. 2011). Bioinformatics analyses on another model organism resulted in the identification of the *sniffer* gene and four additional genes coding for four individual carbonyl reductases, CR1 – CR4, in the *Daphnia* genome (Ebert et al. 2018). We cloned all four *Daphnia* carbonyl reductases from *D. magna* and *D. pulex*, respectively, and showed their high affinity toward aromatic dicarbonyls including redox cyclers such as phenanthrene quinone, implying their physiological role in the detoxification of quinones (Ebert et al. 2018).

Since the mechanism of toxicity and carcinogenicity of TNT and its derivatives occurs through its capability of inducing oxidative stress in the target biota, we had the idea if TNT can induce the gene expression of carbonyl reductase in blue mussels. As a first step, we were able to identify and clone the gene coding for carbonyl reductase in the blue mussel (*Mytilus* spp.) (Fig. 2). The *Mytilus* carbonyl reductase shares 89% identity to human CBR1 and can be regarded as a functional homolog to human CBR1 because it shares all the essential SDR motifs (Persson et al. 2009; Martin et al. 2011). As the second step, blue mussels (*Mytilus* spp.) were exposed to TNT in two laboratory experiments and in a field study and then investigated in terms of their response in form of carbonyl reductase gene induction. As the third step, we determined the occurrence of TNT and derivatives thereof in the mussel tissues and to relate the concentrations of these explosives to the extent of carbonyl reductase gene induction. Our results indeed indicate that carbonyl reductase is induced upon TNT exposure and could possibly serve as the first molecular biomarker for this explosive compound.

Mussels were dissected, and gills, hepatopancreas, mantle and muscle tissue were investigated individually for carbonyl reductase gene expression. Especially in the gills, the respiratory organ with intensive blood circulation and first contact with pollutants from the surrounding water, we found significant correlations between TNT exposure and carbonyl reductase gene induction. This indicates a high sensitivity of carbonyl reductase to TNT exposure. Interestingly, the extent of carbonyl reductase gene induction showed a strong correlation to the initial TNT concentrations in the different aquaria. Likewise, carbonyl reductase gene induction was seen for hepatopancreas as the main digestive and metabolic organ in mussels. Basal carbonyl reductase expression could also be observed in mantle and muscle tissue, but compared to gills and hepatopancreas this expression was much weaker and induction was hardly shown. In general, the metabolic activity in mantle and muscle tissues is relatively low, therefore, they served as a comparison for the tissue-dependent basal and induced expression of carbonyl reductase in our study.

To relate the level of gene induction to the content of TNT in the mussel tissues, we determined the tissue concentrations of TNT and metabolites thereof via GC–MS/MS analysis. Although for the laboratory exposure studies only TNT (2,4,6-trinitrotoluene) itself was dissolved in the aquaria, mussel tissue determination by GC–MS/MS analysis yielded the two TNT metabolites 2-amino-4,6-dinitrotoluene (2-ADNT) and 4-amino-2,6-dinitrotoluene (4-ADNT) as the principal explosives. This finding does also apply for the field experiment. While in mussels placed directly on the ground, TNT itself, as well as 2- and 4-ADNT was observed, in mussels placed one meter above the seabed at the same position, only 4-ADNT but neither TNT nor 2-ADNT could be detected. It is well known, that transformation processes of parent TNT include photolysis, hydrolysis, oxidation, reduction, and biological transformation (Goodfellow et al. 1983). Apparently, the carbon-2 and carbon-4 nitro moieties of TNT had been reduced as a consequence of microbial metabolism or via conversion by mussel enzymes to 2-ADNT and 4-ADNT, respectively (Hawari et al. 2000), both in the lab experiment as well as in the field study.

A very interesting discovery was made during our field experiment at the dumping site Kolberger Heide. Here, the mRNA expression of carbonyl reductase in the mussels directly exposed at the seabed (7 U in Fig. 7) was statistically significant induced (*p* < 0.01) in all analyzed tissues (gills, hepatopancreas and mantle) compared to mussels from one meter above at the same mooring. Although the mussels from 7 U and 7 O were only one meter apart from each other, this difference can be explained with the differing TNT water concentrations pattern in this area. Beck et al. (2019) measured a TNT water concentration of 3.1 mg/L in water samples directly taken at a chunk of hexanite (7 U). In contrast, already at a 50 cm distance only 3.3 µg/L of TNT could be measured in the water samples.

Of note, the TNT concentrations in the water column at the position on the ground (7 U) are more or less comparable to the (higher) concentrations of TNT in the lab studies, indicating that carbonyl reductase gene induction in the field study is indeed a result of the high TNT concentration at this position (7 U). This is supported by the fact that significant carbonyl reductase gene expression occurs already at tissue concentrations below 5 µg of explosives (sum of TNT plus 2-ADNT and 4-ADNT) per g (wet weight) of mussel tissue.

## Conclusions

TNT leaking from dumped conventional munitions poses a major threat to the marine environment (Nipper et al. 2001; Ek et al. 2008). In addition, TNT is known for its mutagenic and carcinogenic effects in humans and may constitute a risk for the human seafood consumer (Bolt et al. 2006; Maser and Strehse 2020). However, measured water and/or tissue concentrations alone are not meaningful in context with TNT effects on an organism. Therefore, a selective biomarker to relate the exposure of an organism to TNT to its pathophysiologic effects on a molecular level is urgently needed. The induced expression of the carbonyl reductase gene from the superfamily of short-chain dehydrogenases/reductases found in the present investigation offers a promising approach as a selective biomarker for environmental pollution caused by TNT.

## References

[CR1] Albertsson E, Larsson DGJ, Förlin L (2010). Induction of hepatic carbonyl reductase/20β-hydroxysteroid dehydrogenase mRNA in rainbow trout downstream from sewage treatment works—possible roles of aryl hydrocarbon receptor agonists and oxidative stress. Aquat Toxicol.

[CR2] Albertsson E, Rad A, Sturve J, Larsson DGJ, Förlin L (2012). Carbonyl reductase mRNA abundance and enzymatic activity as potential biomarkers of oxidative stress in marine fish. Mar Environ Res.

[CR3] Appel D, Strehse JS, Martin H-J, Maser E (2018). Bioaccumulation of 2,4,6-trinitrotoluene (TNT) and its metabolites leaking from corroded munition in transplanted blue mussels (*M. edulis*). Mar Pollut Bull.

[CR4] Beck AJ, Gledhill M, Schlosser C, Stamer B, Böttcher C, Sternheim J, Greinert J, Achterberg EP (2018). Spread, behavior, and ecosystem consequences of conventional munitions compounds in coastal marine waters. Front Mar Sci.

[CR5] Beck AJ, van der Lee EM, Eggert A, Stamer B, Gledhill M, Schlosser C, Achterberg EP (2019). In situ measurements of explosive compound dissolution fluxes from exposed munition material in the Baltic sea. Environ Sci Technol.

[CR6] Bolt HM, Degen GH, Dorn SB, Plöttner S, Harth V (2006). Genotoxicity and potential carcinogenicity of 2,4,6-trinitrotoluene: structural and toxicological considerations. Rev Environ Health.

[CR7] Botella JA, Ulschmid JK, Gruenewald C, Moehle C, Kretzschmar D, Becker K, Schneuwly S (2004). The Drosophila carbonyl reductase sniffer prevents oxidative stress-induced neurodegeneration. Curr Biol CB.

[CR8] Böttcher C, Knobloch T, Rühl N-P, Sternheim J, Wichert U, Wöhler J (2011) Munitionsbelastung der Deutschen Meeresgewässer- Bestandsaufnahme und Empfehlungen (Stand 2011)

[CR9] Cubero-Leon E, Ciocan CM, Minier C, Rotchell JM (2012). Reference gene selection for qPCR in mussel, *Mytilus edulis*, during gametogenesis and exogenous estrogen exposure. Environ Sci Pollut Res.

[CR10] Dawson TM (2003). Molecular pathways of Neurodegeneration in Parkinson’s disease. Science.

[CR11] Doorn JA, Maser E, Blum A, Claffey DJ, Petersen DR (2004). Human carbonyl reductase catalyzes reduction of 4-oxonon-2-enal ^†^. Biochemistry.

[CR12] Ebert B, Kisiela M, Malátková P, El-Hawari Y, Maser E (2010). Regulation of human carbonyl reductase 3 (CBR3; SDR21C2) expression by Nrf2 in cultured cancer cells. Biochemistry.

[CR13] Ebert B, Ebert D, Koebsch K, Maser E, Kisiela M (2018). Carbonyl reductases from Daphnia are regulated by redox cycling compounds. FEBS J.

[CR14] Ek H, Nilsson E, Dave G (2008). Effects of TNT leakage from dumped ammunition on fish and invertebrates in static brackish water systems. Ecotoxicol Environ Saf.

[CR15] Ellis EM (2007). Reactive carbonyls and oxidative stress: potential for therapeutic intervention. Pharmacol Ther.

[CR16] Esterbauer H, Schaur RJ, Zollner H (1991). Chemistry and biochemistry of 4-hydroxynonenal, malonaldehyde and related aldehydes. Free Radic Biol Med.

[CR17] Farrington JW, Tripp BW, Tanabe S, Subramanian A, Sericano JL, Wade TL, Knap AH (2016). Edward D. Goldberg’s proposal of “the Mussel Watch”: reflections after 40 years. Mar Pollut Bull.

[CR18] Gledhill M, Beck AJ, Stamer B, Schlosser C, Achterberg EP (2019). Quantification of munition compounds in the marine environment by solid phase extraction—ultra high performance liquid chromatography with detection by electrospray ionisation—mass spectrometry. Talanta.

[CR19] Goodfellow WL, Burton DT, Graves WC, Hall LW, Cooper KR (1983). Acute toxicity of picirc acid and picramic acid to rainbow trout, Salmo Gairdneri, and American Oyster, Crassostrea Virginica. J Am Water Resour Assoc.

[CR20] Hawari J, Beaudet S, Halasz A, Thiboutot S, Ampleman G (2000). Microbial degradation of explosives: biotransformation versus mineralization. Appl Microbiol Biotechnol.

[CR21] Juhasz AL, Naidu R (2007). Explosives: fate, dynamics, and ecological impact in terrestrial and marine environments. Reviews of environmental contamination and toxicology, reviews of environmental contamination and toxicology.

[CR22] Kampmeier M, van der Lee EM, Wichert U, Greinert J (2020). Exploration of the munition dumpsite Kolberger Heide in Kiel Bay, Germany: example for a standardised hydroacoustic and optic monitoring approach. Cont Shelf Res.

[CR23] Koske D, Goldenstein NI, Kammann U (2019). Nitroaromatic compounds damage the DNA of zebrafish embryos (*Danio rerio*). Aquat Toxicol.

[CR24] Koske D, Straumer K, Goldenstein NI, Hanel R, Lang T, Kammann U (2020). First evidence of explosives and their degradation products in dab (*Limanda limanda* L.) from a munition dumpsite in the Baltic Sea. Mar Pollut Bull.

[CR25] Liao H-Y, Kao C-M, Yao C-L, Chiu P-W, Yao C-C, Chen S-C (2017). 2,4,6-Trinitrotoluene induces apoptosis via ROS-regulated mitochondrial dysfunction and endoplasmic reticulum stress in HepG2 and Hep3B cells. Sci Rep.

[CR26] Lotufo GR, Belden JB, Fisher JC, Chen S-F, Mowery RA, Chambliss CK, Rosen G (2016). Accumulation and depuration of trinitrotoluene and related extractable and nonextractable (bound) residues in marine fish and mussels. Environ Pollut.

[CR27] Lushchak VI (2011). Environmentally induced oxidative stress in aquatic animals. Aquat Toxicol.

[CR28] Martin H-J, Ziemba M, Kisiela M, Botella JA, Schneuwly S, Maser E (2011). The Drosophila carbonyl reductase sniffer is an efficient 4-oxonon-2-enal (4ONE) reductase. Chem Biol Interact.

[CR29] Maser E (2006). Neuroprotective role for carbonyl reductase?. Biochem Biophys Res Commun.

[CR30] Maser E, Strehse JS (2020). “Don’t Blast”: blast-in-place (BiP) operations of dumped World War munitions in the oceans significantly increase hazards to the environment and the human seafood consumer. Arch Toxicol.

[CR31] Miura T, Taketomi A, Nishinaka T, Terada T (2013). Regulation of human carbonyl reductase 1 (CBR1, SDR21C1) gene by transcription factor Nrf2. Chem Biol Interact.

[CR32] Naumenko EA, Ahlemeyer B, Baumgart-Vogt E (2017). Species-specific differences in peroxisome proliferation, catalase, and SOD2 upregulation as well as toxicity in human, mouse, and rat hepatoma cells induced by the explosive and environmental pollutant 2,4,6-trinitrotoluene: species-specific differences to TNT-induced oxidative stress. Environ Toxicol.

[CR33] Nipper M, Carr RS, Biedenbach JM, Hooten RL, Miller K, Saepoff S (2001). Development of marine toxicity data for ordnance compounds. Arch Environ Contam Toxicol.

[CR34] OECD (2009). Test No. 230: 21-day fish assay: a short-term screening for oestrogenic and androgenic activity, and aromatase inhibition, OECD guidelines for the testing of chemicals, section 2. OECD.

[CR35] OECD (2019). Test No. 203: fish, acute toxicity test, OECD guidelines for the testing of chemicals, section 2. OECD.

[CR36] Penning TM (2015). The aldo-keto reductases (AKRs): overview. Chem Biol Interact.

[CR37] Persson B, Kallberg Y, Bray JE, Bruford E, Dellaporta SL, Favia AD, Duarte RG, Jörnvall H, Kavanagh KL, Kedishvili N, Kisiela M, Maser E, Mindnich R, Orchard S, Penning TM, Thornton JM, Adamski J, Oppermann U (2009). The SDR (short-chain dehydrogenase/reductase and related enzymes) nomenclature initiative. Chem Biol Interact.

[CR38] Porter JW, Barton JV, Torres C, Machlis GE, Hanson T, Špirić Z, McKendry JE (2011). Ecological, radiological, and toxicological effects of naval bombardment on the Coral Reefs of Isla de Vieques, Puerto Rico. Warfare ecology.

[CR39] Rosen G, Lotufo GR (2007). Toxicity of explosive compounds to the marine mussel, *Mytilus galloprovincialis*, in aqueous exposures. Ecotoxicol Environ Saf.

[CR40] Sayre L, Smith M, Perry G (2001). Chemistry and biochemistry of oxidative stress in neurodegenerative disease. Curr Med Chem.

[CR41] Schuster R, Strehse JS, Ahvo A, Turja R, Maser E, Bickmeyer U, Lehtonen KK, Brenner M (2020) Exposure to dissolved TNT causes multilevel biological effects in Baltic mussels (*Mytilus* spp.). under review in Marine Pollution Bulletin 10.1016/j.marenvres.2021.105264 33725510

[CR42] Strehse JS, Maser E (2020). Marine bivalves as bioindicators for environmental pollutants with focus on dumped munitions in the sea: a review. Mar Environ Res.

[CR43] Strehse JS, Appel D, Geist C, Martin H-J, Maser E (2017). Biomonitoring of 2,4,6-trinitrotoluene and degradation products in the marine environment with transplanted blue mussels (*M. edulis*). Toxicology.

[CR44] Stuckas H, Knöbel L, Schade H, Breusing C, Hinrichsen H-H, Bartel M, Langguth K, Melzner F (2017). Combining hydrodynamic modelling with genetics: can passive larval drift shape the genetic structure of Baltic *Mytilus* populations?. Mol Ecol.

[CR45] Watanabe K, Sugawara C, Ono A, Fukuzumi Y, Itakura S, Yamazaki M, Tashiro H, Osoegawa K, Soeda E, Nomura T (1998). Mapping of a novel human carbonyl reductase, CBR3, and ribosomal pseudogenes to human chromosome 21q22.2. Genomics.

[CR46] Wermuth B (1981). Purification and properties of an NADPH-dependent carbonyl reductase from human brain. Relationship to prostaglandin 9-ketoreductase and xenobiotic ketone reductase. J Biol Chem.

