# Correction to: The explosive trinitrotoluene (TNT) induces gene expression of carbonyl reductase in the blue mussel (*Mytilus* spp.): a new promising biomarker for sea dumped war relicts?

**DOI:** 10.1007/s00204-021-03088-y

**Published:** 2021-06-20

**Authors:** Jennifer S. Strehse, Matthias Brenner, Michael Kisiela, Edmund Maser

**Affiliations:** 1 Institute of Toxicology and Pharmacology for Natural Scientists, University Medical School Schleswig-Holstein, Brunswiker Str. 10, 24105 Kiel, Germany; 2 Alfred Wegener Institute Helmholtz Centre for Polar and Marine Research, Am Handelshafen 12, 27570 Bremerhaven, Germany

## Correction to: Archives of Toxicology (2020) 94:4043–4054 10.1007/s00204-020-02931-y

The article “The explosive trinitrotoluene (TNT) induces gene expression of carbonyl reductase in the blue mussel (Mytilus spp.): a new promising biomarker for sea dumped war relicts?”, written by Jennifer S. Strehse, Matthias Brenner, Michael Kisiela, and Edmund Maser, was originally published Online First without Open Access. After publication in volume 94, issue 12, page 4043–4054 the author decided to opt for Open Choice and to make the article an Open Access publication. Therefore, the copyright of the article has been changed to © The Author(s) 2020 and the article is forthwith distributed under the terms of the Creative Commons Attribution 4.0 International License, which permits use, sharing, adaptation, distribution and reproduction in any medium or format, as long as you give appropriate credit to the original author(s) and the source, provide a link to the Creative Commons licence, and indicate if changes were made. The images or other third party material in this article are included in the article’s Creative Commons licence, unless indicated otherwise in a credit line to the material. If material is not included in the article’s Creative Commons licence and your intended use is not permitted by statutory regulation or exceeds the permitted use, you will need to obtain permission directly from the copyright holder. To view a copy of this licence, visit http://creativecommons.org/licenses/by/4.0. Open access funding enabled and organized by Projekt DEAL.

Original article has been corrected.

